# Massive Open Online Courses in Public Health

**DOI:** 10.3389/fpubh.2013.00059

**Published:** 2013-11-25

**Authors:** Ira Gooding, Brian Klaas, James D. Yager, Sukon Kanchanaraksa

**Affiliations:** ^1^Center for Teaching and Learning, Johns Hopkins Bloomberg School of Public Health, Baltimore, MD, USA; ^2^Department of Environmental Health Sciences, Johns Hopkins Bloomberg School of Public Health, Baltimore, MD, USA; ^3^Department of Epidemiology, Johns Hopkins Bloomberg School of Public Health, Baltimore, MD, USA

**Keywords:** massive open online courses, MOOCs, online education, public health, technology

## Abstract

Massive open online courses (MOOCs) represent a new and potentially transformative model for providing educational opportunities to learners not enrolled in a formal educational program. The authors describe the experience of developing and offering eight MOOCs on a variety of public health topics. Existing institutional infrastructure and experience with both for-credit online education and open educational resources mitigated the institutional risk and resource requirements. Although learners are able to enroll easily and freely and do so in large numbers, there is considerable variety in the level of participation and engagement among enrollees. As a result, comprehensive and accurate assessment of meaningful learning progress remains a major challenge for evaluating the effectiveness of MOOCs for providing public health education.

## Introduction

Massive open online courses (MOOCs) have emerged as an important and perhaps transformative development in higher education. The Johns Hopkins Bloomberg School of Public Health (JHSPH) has chosen to be part of this development by offering several MOOCs on public health topics during its 2012–2013 academic year. During that time, JHSPH used its existing resource and policy infrastructure to quickly implement MOOCs and to begin exploring what they have to offer public health learners, instructors, and the university.

## The Emergence of MOOCs

Massive open online courses first started to emerge several years ago as educators began experimenting with the open educational resources (OERs) introduced through a variety of ventures like OpenCourseWare (OCW) and similar sites that provide openly licensed and adaptable teaching materials. These resources were combined with advances in online communication and collaboration technology to create the first MOOCs. The term MOOC was initially used to describe George Siemens’ and Stephen Downes’ “Connectivism and Connective Knowledge” course in 2008 and 2009 as well as courses taught by David Wiley, Alec Couros, and Jim Groom (1).

These connectivist MOOCs (cMOOCs) were built around core open content that learners were invited to consume and react to, but the heart of a cMOOC is the connected collective of learners and their individual contributions to the teaching and learning enterprise (2). As a result, the cMOOCs represent the most truly open manifestation of the MOOC phenomenon; they employ open content *and* invite participants to engage in the practical openness of developing, directing, shaping, and ultimately teaching the course as it happens. Interesting examples of ongoing cMOOCs are DS106: Digital Storytelling^1^ and Open Course in Technology Enhanced Learning^2^.

Many learners and educators recognize cMOOCs as interesting and productive learning experiments and experiences, but they don’t necessarily recognize cMOOCs as courses because they don’t look or feel like any course they’ve ever taken or taught. Many excellent students and outstanding teachers think of a course as far more highly structured than a true cMOOC can allow itself to be. As rewarding as the practice of cMOOC openness is for some, there are many learners, particularly those living with constraints of time and resources, who prefer structured transfer of knowledge in a conventional course framework. Further, elite research institutions like MIT, which had led the way with OCW, and Stanford recognized an opportunity to scale up the MOOC concept with institutional support. Hence, the emergence of xMOOCs.

Although the definition is still under debate and will likely be so far some time, an xMOOC is generally more conventionally course-like than a cMOOC. They usually feature a structured sequence of lectures, exercises, assessments, and communication opportunities. An xMOOC also usually features a clear delineation of teachers and learners. xMOOCs also scale far more easily than cMOOCs.

When Daphne Koller, Andrew Ng, and Sebastian Thrun launched their Stanford MOOCs in the autumn of 2011, the enrollment in each exceeded 100,000 students (3). Sebastian Thrun’s Artificial Intelligence course taught with Google’s Peter Norvig had 160,000 registered students, and the Machine Learning Course taught by Daphne Koller and Andrew Ng registered 104,000 students (4). The sheer scale captured the imagination of many, both inside the academy and out. With the blinding speed of innovation, three startups emerged from Stanford: Udacity, Udemy, and Coursera. Not to be outdone, MIT announced its forthcoming MITx platform, which would soon morph into EdX as new partners like Harvard came aboard.

Coursera initially partnered with four founding universities, Stanford University, University of Pennsylvania, University of Michigan, and Princeton University, and then set about inviting other elite research universities to join. Among the new Coursera partners announced in the summer of 2012 was Johns Hopkins University, particularly the Bloomberg School of Public Health.

## Why JHSPH Started Offering MOOCs

JHSPH has been offering online courses since 1997. As of 2013, it offers 113 fully online courses for credit^3^, and students can earn a Masters of Public Health (MPH) degree through the School’s Part-time/Online MPH program, as well as other Masters and Doctoral degrees and certificates.

Johns Hopkins Bloomberg School of Public Health joined the OER movement in 2005 when it launched JHSPH OCW^4^ as an effort to share the School’s extensive collection of teaching materials from diverse public health discipline areas with independent learners and educators. Since then, JHSPH OCW has grown to include materials from more than 110 academic courses, symposia, and training programs. Users include public health professionals seeking to refresh their knowledge, educators who adopt or adapt teaching materials in their courses, and students at JHSPH and elsewhere who are planning their course of study. The materials posted on OCW may be used as posted or adapted for reuse under the terms of the Creative Commons Attribution-Non-Commercial-ShareAlike license^5^.

Both the online academic courses and JHSPH OCW are coordinated and supported by the JHSPH Center for Teaching and Learning (CTL). Staffed by instructional designers, web developers, audio producers, and technical writers, CTL provides a crucial infrastructure to support the world-class researchers and educators at JHSPH.

The School’s extensive experience with both online and open education and the infrastructure provided by CTL made the decision to begin offering MOOCs a relatively easy one.

Johns Hopkins Bloomberg School of Public Health is committed to pursuing both of its core enterprises, discovery and education, on a global scale. Online learning and open education have provided the tools for offering a range of options to people interested in learning about public health. At one end of the spectrum, JHSPH offers on campus and online courses for academic credit. At the other end of the spectrum are self-guided OER opportunities freely available on demand at JHSPH OCW. MOOCs fit neatly between the two ends of this spectrum. Like many institutions, JHSPH sees MOOCs as an important development that will help it achieve its educational mission by providing online learning opportunities with no financial barriers to entry and built largely on existing OER that are also more guided, participatory, and engaging than the raw OER content available on JHSPH OCW.

Some in academia are understandably concerned about the possibility that MOOCs will undermine the existing university business model (5) or undermine the professional autonomy of university instructors (6). Universities are reliant on tuition revenue, and the suggestion that MOOCs can be offered and taken for free has the potential to undermine the model in four distinct ways. First, students might elect to take free MOOCs and forego for-credit offerings that require tuition payment. Second, offering MOOCs for free might send the counterproductive message that a university’s offerings are not sufficiently valuable to justify existing tuition. Third, competitor institutions may package several MOOCs for a nominal fee and grant credentials. Fourth, MOOCs could be accepted for credit by institutions that will no longer need to employ instructors.

Although it is too soon to know for sure how MOOCs will affect enrollment in conventional in-person or online university courses, JHSPH is drawing on its experience with OCW to inform its decision to offer MOOCs. An analysis of 24 online for-credit JHSPH courses with materials published on OCW demonstrated that there was no significant change in course enrollment in the aftermath of publication on OCW (7). In fact, enrollment in online courses at JHSPH, of which 27% have their content published on OCW, continues to increase.

In addition to the analysis of actual enrollment, JHSPH has also measured the influence of OCW on enrollment decisions among JHSPH students. A multi-year survey of 1043 JHSPH students revealed that OCW positively influenced the decision to attend JHSPH in 72 (11.75%) of the 613 JHSPH students who were aware of OCW’s existence. In addition, 56 (70%) of 80 students who reported using OCW for academic planning indicated that the materials on OCW positively influenced their course selection decisions (7).

During the 2012 admissions cycle, JHSPH added questions specifically about OCW to the JHSPH admissions application to gauge both awareness and influence of OCW. The survey found that 39% of the School’s 3,905 applicants were aware of OCW before completing their admissions application. It also revealed that 25% of all applicants reported that OCW was influential in their decision to apply to JHSPH.

These measures of open education’s impact on enrollment in conventional courses and degree programs indicate that JHSPH has not experienced a negative impact on its enrollment numbers or the perceived value of its conventional programs. If anything, open education has enhanced the reputation of JHSPH, making it more widely known and attractive to those who are able and ready to pursue formal course work. JHSPH started publishing OCW as a way to share its resources with people who might not otherwise have access to it and has now found that its already positive reputation is burnished by sharing. This is a classic illustration of an institution doing well by doing good, and it is this experience that is informing the administration of JHSPH in its decisions about MOOCs.

The JHSPH experience with OCW also laid the intellectual property and copyright groundwork for offering MOOCs. Due to its experience with OCW, JHSPH had already instituted rules for content ownership and had nurtured a culture of openness prior to being approached by Coursera. As a result, complicated debates about intellectual property did not emerge during the deliberations about offering MOOCs, freeing the institution to focus on matters of pedagogy, technical feasibility, and logistics. As a matter of policy, faculty at JHSPH have always been free to participate in OCW or not; no faculty members are compelled to participate against their will. The same approach is taken with MOOCs, and the experience with OCW has contributed to a high degree of comfort among the faculty.

Admittedly, the OCW model is sufficiently different from the MOOC model to warrant caution against drawing unjustified conclusions. However, the waters are uncharted, and uncharted waters call for reliance on experience and vision. Going forward, JHSPH will collect data on the impact of MOOCs on conventional enrollment to discern whether its experience with OCW is a reliable guide.

## The First Johns Hopkins MOOCs

Eight initial courses were announced when Coursera and JHU announced their partnership in summer of 2012. All eight of these MOOCs originated from within JHSPH and were taught by JHSPH faculty. Two courses were launched in September 2012, three more in October 2012, and three more in January 2013. One of the MOOCs first offered in September 2012 (*Computing for Data Analysis*) was also offered for a second time in January 2013, for a total of nine MOOC offerings over the course of 6 months.

Most of the faculty members who taught the first eight JHSPH MOOCs were recruited by the administration and CTL because of their mission-based approach to education and their experience with online teaching. JHSPH is home to many centers, institutes, and individual faculty members who have a powerful commitment to broad dissemination of the public health education, and the School chose to leverage this commitment to get its MOOC efforts off the ground. By focusing its recruitment efforts on mission-driven faculty with online teaching experience, five MOOC offerings were quickly identified. In addition to these faculty members who were actively recruited, three other instructors without online teaching experience also volunteered to develop courses, bringing the inaugural slate of JHSPH MOOCs to eight.

## MOOC Development Resource Requirements

Johns Hopkins Bloomberg School of Public Health has relied primarily on its existing online course development and production infrastructure to offer MOOCs on Coursera. In addition, recorded content from existing online courses has been adapted for use in MOOCs. As a result, the efforts at JHSPH have been less resource- and labor-intensive than is likely required for efforts of similar scale at other universities without existing infrastructure and recorded content.

A single full-time member of the CTL team oversees the development and deployment of all JHSPH MOOCs. Additional staff, such as instructional designers, technical writers, and audio producers, and resources, such as recording facilities and instructional technology, are drawn upon as needed during both the development and deployment stages. However, the level of involvement varies depending on the course and the preferences of the instructors.

At one end of the spectrum, a minimal level of development and administrative support was provided to those instructors who felt comfortable working independently with the Coursera platform and with desktop recording and editing equipment. These instructors assumed personal responsibility for most of their own development and production work, and called upon CTL and institutional resources only when necessary.

At the other end of the spectrum, a great deal of CTL support was provided to some other MOOC instructors. Some requested instructional design consultations to plan learning activities and develop assessment strategies. Some requested production support to record new video content that was not already on hand from existing online courses. Some requested training and support for using the Coursera platform. CTL maintained a flexible approach, knowing that each individual instructor would have different requirements.

Some instructors also relied on additional support from teaching assistants (TAs) funded by JHSPH administration through the instructor’s respective departments. In most cases, the TAs supported the faculty during the offering by participating in the online discussion forums, answering student questions in some cases and funneling interesting forum activity to faculty in others. As in the use of CTL resources, each instructor’s use of TA support was left to their own discretion.

The variety of approaches taken at JHSPH makes it very difficult to characterize a typical MOOC development experience or to quantify the resources required. Nevertheless, development of a MOOC at JHSPH has required anywhere from 30 to 80 h of instructor/TA time and between 10 and 40 h of CTL support time.

## MOOC Offering Resource Requirements

Assuming that all of the lectures, assessments, and other materials have been posted by the time a course offering begins, the work during the course offering mainly consists of communication with students on the discussion forums and through e-mail announcements and troubleshooting technical and logistical problems that emerge from time to time.

At JHSPH, a member of the CTL team monitors the discussion forums on a daily basis and responds to students’ logistical and technical questions. The knowledge that these matters are being handled by CTL frees the instructor to focus their energy on responding to questions and comments about the course topics. There are a variety of strategies that instructors can use to handle this component of the MOOC teaching experience. The following approaches have been used by instructors at JHSPH.

### Participation in organic discussions

In this model, the instructor actively participates in the discussion forums but students are leading the conversations by posing questions, commenting on the materials, and helping one another. The instructor mostly reacts to the students’ posts and comments that emerge organically as the students work their way through the course. The instructor monitors the discussions on a daily basis and responds as needed. This approach requires little planning and empowers the learners in a style that is more akin to cMOOCs. Students are presented with a core set of instructional materials and then invited into a conversation that they can then take in any direction that they choose. This approach can be very rewarding for students, but its unpredictability makes it challenging for some instructors. It can also be very time consuming because the wide variety of questions and comments posted by students makes it difficult for instructors to decide when and how to respond. Another downside of this approach is that many students might not participate in the forums unless the instructor asks them to respond to a specific topic or question.

### Leading planned discussions

In this model, the instructor guides the discussion forum activity by posing questions, assigning discussion exercises, and sometimes steering organically emerging conversations toward course learning objectives. By planning discussions in advance, instructors can participate in a more intentional and predictable manner. When using this model, it is important to set clear expectations so that students understand the instructor’s strategy. Although this approach to the forums is time consuming during the development phase, it can lead to a less taxing experience during the course offering. The most prominent drawback to this approach is that students may feel less empowered to ask their own questions or share their own insights. To minimize this problem, planned discussions should be presented as an invitation to a focused conversation but without unnecessary boundaries, due dates, or other limitations. Another way to keep planned discussions from squelching organically emerging discussions is to create a Student’s Forum in which students can begin and participate in their own discussions without worrying about going off topic.

### Mediated participation

In this model, instructors do not participate in regular direct interaction on the discussion forums. Instead, TAs or other staff monitor and participate in the forums on a daily basis and gather interesting questions and comments for the instructor. At regular intervals, the instructor then responds in a collective message with their own thoughts and reactions. This response can be made in the form of an announcement that appears on the course home page and is sent by e-mail to the whole class or in the form a video response that is posted for students to view. This strategy allows busy instructors who are not able to participate on a daily basis to still have a presence in the ongoing conversations that emerge organically on the discussion forums. The most significant drawback to this approach is that the instructor is both spatially and chronologically remote from the ongoing conversations. They are spatially remote because their responses are not posted within the forums where the rest of the conversation occurs, and they are chronologically remote because the comments may come days after the conversation was at its peak.

Similar to the case with course development, the variety of approaches taken at JHSPH makes it difficult to characterize a typical MOOC offering experience or to quantify the resources required to support students during an offering. MOOC offerings at JHSPH have required anywhere from 1 to 5 h of instructor/TA time per week and between 5 and 10 h of CTL support time per week.

## Enrollment in JHSPH MOOCs

Massive open online course enrollments are continually in flux, with students joining and leaving at will. As a result, enrollment totals can vary depending on the time point of record. At JHSPH, we have chosen the moment of final grade calculation soon after the end of the course (EOC) as the point at which we capture official EOC enrollment data.

**Figure 1:**
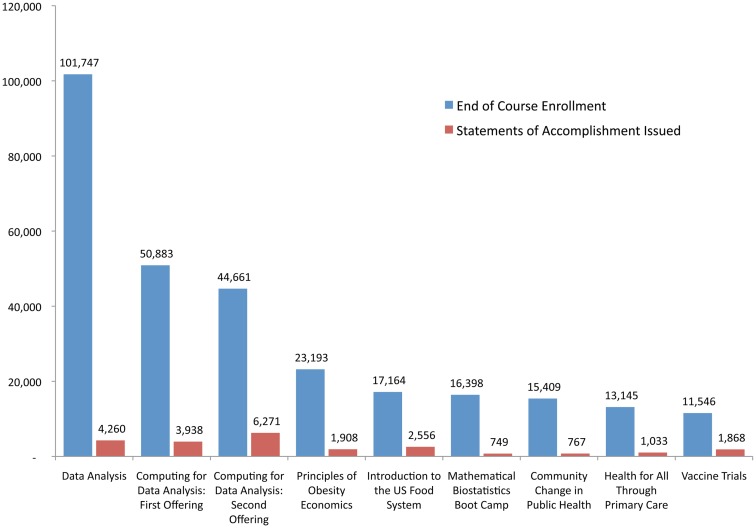
**End of course enrollment and statements of accomplishment issued in nine initial JHSPH MOOC offerings**.

Total EOC enrollment in the nine JHSPH MOOCs offered to date was 294,146 learners. EOC enrollments have ranged from 11,546 learners enrolled in *Vaccine Trials: Methods and Best Practices* to 101,747 learners enrolled in *Data Analysis*. The median EOC enrollment in JHSPH MOOC offerings is 17,164. Figure 1 shows the EOC enrollment for all eight initial course offerings and for the second offering of *Computing for Data Analysis*.

The learners who have enrolled in JHSPH MOOC offerings come from all over the world. Surveys of 10,668 learners enrolled in three JHSPH MOOCs (*Vaccine Trials, Community Change in Public Health*, and *Mathematical Biostatistics Boot Camp*) indicate that 43% of enrollees are from North America, and 22% are from Europe, 18% are from Asia, 7% are from Africa, 7% are from South America, and 3% are from Australia/Oceania.

## Student Participation in JHSPH MOOCs

Although assessment methods and grading policies vary from MOOC to MOOC, students in all nine of these initial JHSPH MOOC offerings have had the opportunity to earn a Statement of Accomplishment (SOA) issued by Coursera and signed by the course instructor. A total of 23,350 SOAs were issued in the first nine JHSPH MOOC offerings, ranging from 749 SOAs in *Mathematical Biostatistics Boot Camp* to 6,271 SOAs in the second offering of *Computing for Data Analysis* (Figure 1). The median number of SOAs issued in JHSPH MOOC offerings is 1,908.

Not every student who enrolls in a MOOC does so with the intention of earning an SOA. According to pre-course surveys administered to enrollees in nine JHSPH MOOC offerings, 18% of enrollees indicated that they planned to only watch lecture videos without participating in the assessment components of the course. It is, therefore, important to examine levels of participation in different course activities besides just course enrollment and SOA achievement.

The 294,146 enrollees can be categorized by their participation in different learning activities within a course. Enrollees who watched or downloaded at least one lecture video are categorized as video participants. A total of 147,187 enrollees (50.3%) were video participants, and the median number of video participants was 8,164 (47.6% of median enrollment).

Enrollees who submitted answers to at least one quiz (the only assessment method employed in all nine offerings) are categorized as quiz participants. A total of 66,771 enrollees (22.7%) were quiz participants, and the median number of quiz participants was 4,450 (25.9% of median enrollment).

Enrollees who posted at least one time on the Discussion Forum are categorized as forum participants. A total of 18,356 enrollees (6.2%) were forum participants, and the median number of forum participants was 1,680 (9.8% of median enrollment).

The dramatic fall-off in each successive step of deepening activity is consistent with the funnel of participation phenomenon that has emerged throughout the world of MOOCs (8). The funnel of participation is based on a marketing concept that categorizes consumers on the basis of their relationship to a product (awareness, interest, desire, and purchase). In the MOOC setting, the funnel categorizes learners in terms of their relationship to a learning activity (awareness, enrollment, activity, progress).

**Figure 2:**
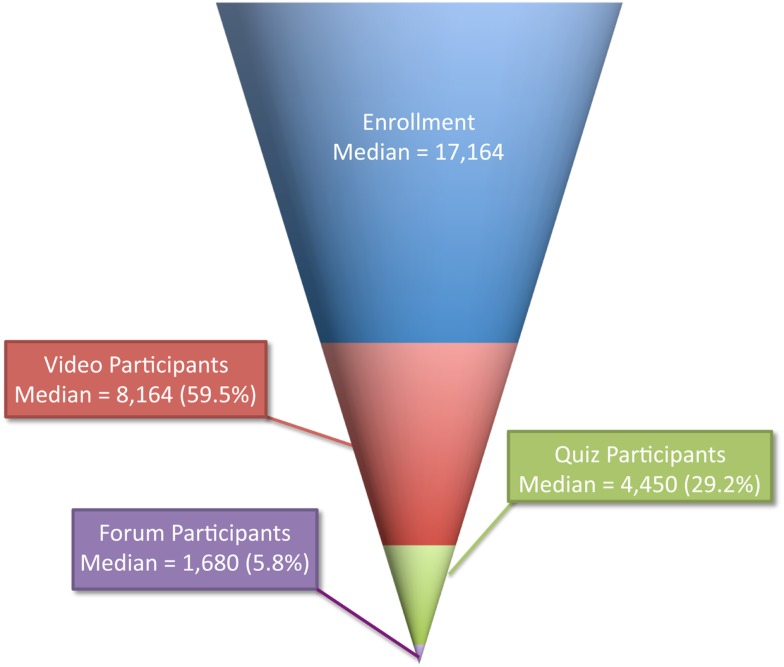
**Funnel of participation in nine JHSPH MOOC offerings**.

There was a clear funnel of participation in the first nine JHSPH MOOC offerings, and the sequence of steps ordered by level of participation is consistent across all nine offerings (Figure 2). Every offering had more video participants than quiz participants and more quiz participants than forum participants. It is important to note that, with the exception of enrollment, participation in any given level is not a prerequisite for participation in any other. For example, an individual student may choose to be a discussion forum participant without being a video or quiz participant. Therefore, each level of participation should be viewed as a subset of the enrollment level and not as a subset of any other level.

## Comparing Student Participation Across JHSPH MOOCs

**Figure 3:**
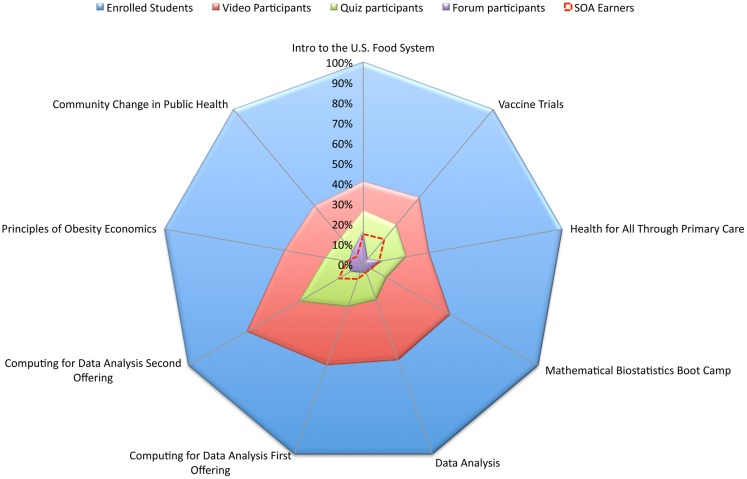
**Looking down the funnel of participation in nine JHSPH MOOC offerings**.

When the funnel of participation of each individual course is examined, some variation emerges (Figure 3). There is a higher rate of video participation in the MOOCs that cover quantitative topics (*Computing for Data Analysis, Data Analysis*, and *Mathematical Biostatistics Boot Camp*) than in the other MOOC offerings, which cover non-quantitative topics. Given the higher rate of video participation, one might expect to see a steeper fall-off between video participation and quiz participation in the quantitative courses, but that is not the case; both types of courses saw about half as many quiz participants as video participants. Participation in the quantitative courses dropped from 59.5% video participation to 29.2% quiz participation; whereas participation in the non-quantitative courses dropped from 37.8% in the 19.1%, respectively.

Another notable point of difference among courses is the rate of forum participation. Compared with the other JHSPH MOOCs, *Introduction to the U.S. Food System* had a relatively high rate of activity on the forums, with 16.6% of enrolled students participating. This particular MOOC had a highly planned forum strategy in comparison with the other JHSPH MOOCs. During each week of the course, the students were given suggested topics of conversation and encouraged to answer questions on the forums. Interestingly, the instructors in this course had very little direct forum interaction. Instead of personally posting on the forums, instructor forum participation was mediated by TAs who gathered interesting posts and discussion threads throughout the week. The instructors then responded via a weekly video post recorded in their office. Despite the lack of direct instructor interaction, the rate of forum participation in this MOOC is a testament to the power of a planned forum strategy.

**Figure 4:**
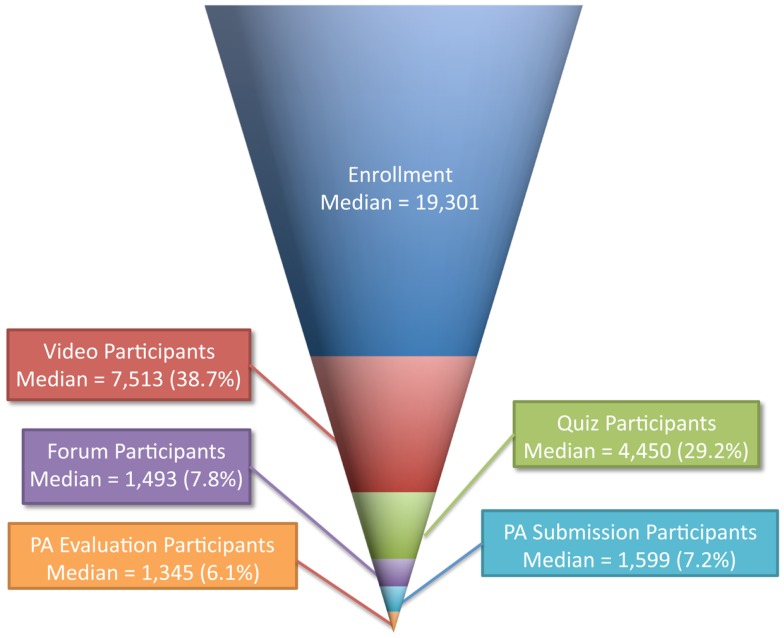
**Funnel of participation in four JHSPH MOOC offerings with peer assessment assignments**.

Some of the MOOCs offered by JHSPH include assessments that go beyond automatically graded multiple choice quizzes. *Computing for Data Analysis* includes programing assignments, and four MOOCs (*Data Analysis, Community Change in Public Health, Health for All Through Primary Care*, and *Principles of Obesity Economics*) feature peer-assessed writing assignments. The funnel of participation for these four courses looks slightly different; there are two levels that are deeper than forum participation: Peer Assessment Submission Participation (i.e., submitting a written answer to the assignment) and Peer Assessment Evaluation Participation (i.e., evaluating the written submissions of other students). The grades of those students who do not perform a stipulated number of evaluations are penalized; nevertheless, there is still a fall-off of participation in the move from Submission Participation to the deeper level of Evaluation Participation (Figure 4). Unlike the relationships among other participation levels, the ability to participate in an assignment’s evaluation is contingent upon participation in the submission phase.

## Meaningful Learning Progress in JHSPH MOOCs

Despite the ease of tracking the varying degrees of participation within MOOCs, the degree of meaningful learning progress is harder to measure. As Clow describes the funnel of participation, meaningful learning progress is the deepest level with the fewest number of participants (8). The group of students who make meaningful learning progress is a subset of the group that participates in some level of activity (video, quiz, forum participation).

One way to measure the size of this group is to look at the rate of SOAs earned. In nine JHSPH MOOC offerings, a median number of students who earned an SOA was 1,908 (11.1%), which is lower than the median number of video participants (8,164; 47.6%) and the median number of quiz participants (4,450; 25.9%) but higher than the median number of forum participants (1,680; 9.8%). Of course, reliance on the rate of SOA issuance to quantify meaningful learning progress assumes the validity of the assessments used, a matter that has yet to be adequately demonstrated.

There is a high degree of variability in the rate of SOA issuance and perhaps a correlation of that rate with assessment modality and deadline flexibility. Of the nine JHSPH MOOC offerings, three used quizzes as the only assessment mode. The median rate of SOA issuance in these courses was 14.9% compared with 7.9% in the MOOCs that combined quiz assessments with other modes like peer assessment and programing assignments. Among the MOOCs with quizzes only, one (*Mathematical Biostatistics Boot Camp)* had firm weekly quiz deadlines and two (*Vaccine Trials* and *Introduction to the U.S. Food System*) had flexible deadlines. The rate of SOA issuance in the MOOC with firm deadlines was 4.6%, and the median rate in the MOOCs with flexible deadlines 15.5%. More data need to be collected on the correlation of student performance with assessment mode and deadline flexibility before generalizable conclusions can be drawn.

Until the validity of MOOC assessments can be established, our only measure of meaningful learning progress is the students’ own perception of learning. Post-course surveys are administered to students in JHSPH MOOCs to gather feedback about the learning experience and their impressions of the course. Across the nine JHSPH MOOC offerings, a total of 10,365 students who earned an SOA and 3,901 students who did not earn an SOA responded to the post-course survey (response rates: earners, 44.4%; non-earners, 1.4%).

When asked whether they found the course useful, 91.2% of respondents who had not earned an SOA selected “Yes, I’ve already learned a lot and feel like it was a good use of my time even if I go no further.” This result indicates that the proportion of enrolled students making meaningful learning progress is greater than just the proportion who earned an SOA.

## Bringing the MOOC Experience Back to Campus

The MOOC experience at Johns Hopkins has not only allowed us to bring our experience to the world, it has changed the way that we think about public health education on campus.

Peer assessments managed in a manner similar to those found on the Coursera platform are an area of intense interest by a number of faculty on campus. While peer assessments have been performed in a mostly manual way in a select number of on-campus courses in the past, the School’s experience with MOOCs has pushed faculty to consider how to create a similar, system-mediated peer assessment process on campus. The CTL is currently building a peer assessment and rubric grading module for the School’s learning management system. Numerous faculty and members of the instructional design team have indicated that they will incorporate this peer assessment tool into their classes in the upcoming academic year.

The use of screencasting technology has seen a sharp uptake since the initial launch of courses on the Coursera platform. Faculty in the department of Biostatistics, early adopters of screencasting tools to create their MOOC lecture videos, have been particularly enthusiastic about the use of screencasting tools in their own on-campus teaching and educational activities.

Finally, the use of in-lecture quizzes in the Coursera platform has sparked strong interest by Bloomberg School of Public Health faculty in having the same capability available in the learning management platform used by the School. Research suggests that in-lecture quizzes aid in student engagement and retention of information in online lectures (9). The School plans on making in-lecture quizzes a part of its learning management system in 2014.

## Conclusion

There is great promise in MOOCs as a new way to provide quality public health educational opportunities to a massive global audience, and much has been learned from the initial JHSPH experience with MOOCs. The faculty and institutional resources required to develop and deploy MOOCs have not been overwhelming when considered in light of their reach and potential impact, but a formal cost-effectiveness analysis is required to confirm this impression.

There is also much work to be done to improve the teaching, knowledge assessment, and measurement of learning progress in MOOCs. Measuring the validity of reliability of MOOC knowledge assessments will be an important step in this process, as will developing methods of heightening and sustaining learner engagement and measuring genuine progress toward learning objectives throughout the funnel of participation.

## Conflict of Interest Statement

The authors are employed by the Johns Hopkins University, which has a partnership agreement with Coursera, a provider of massive open online courses. The authors receive no direct financial benefit from this partnership agreement.

## Footnotes

^1^http://ds106.us/

^2^http://octel.alt.ac.uk/

^3^http://distance.jhsph.edu/

^4^http://ocw.jhsph.edu

^5^http://creativecommons.org/
